# Supplementary material for: Overexpression of LAR1 Suppresses Anthocyanin Biosynthesis by Enhancing Catechin Competition Leading to Promotion of Proanthocyanidin Pathway in Spine Grape (Vitis davidii) Cells
Source: Int J Mol Sci. 2024 Nov 11;25(22):12087. doi: 10.3390/ijms252212087 (PMC11594265; doi:10.3390/ijms252212087)
Supplement: Supplementary file 1 [file ijms-25-12087-s001.zip › ijms-3260132-supplementary.pdf]

Supplementary Table

Table S1. The primers used in this study.

Primer	Sequence (5'-3')	Note
VdLAR1-F	TCCATAACTCCGTGTGCTGT	Homologous cloning of *VdLAR1*
VdLAR1-R	AGGCAATCAAGCAAGTTCCC	
VdLAR1-BamHI-F	GAACACGGGGGACTCTAGAGATGACTGTTTCTCCGGTTCC	Construction of overexpression vector
VdLAR1-XmaI-R	ACCATGGTGGCGACCGGTACAGCGCAGGTTGCAGTGACTT	
pBI121-F	TCCTTCGCAAGACCCTTCCTCT	Transgenic lines identification
pBI121-R	GAACTTGTGGCCGTTTACGTCG	
VdDFR-F	TGCTGATGAAGGAAGTTTCG	qRT-PCR analysis
VdDFR-R	GCGGAGGATGTGAATACAAG	
VdLDOX-F	GGAAGTTGGTGGGATGGA	
VdLDOX-R	GTGGAGGATGAAGGTGAGAG	
VdLAR1-F	GGTTTCATTGGTCAGTTCGT	
VdLAR1-R	GACTCCTGCTCGTTTATCA	
VdLAR2-F	CAATACCCACCCTGCTGA	
VdLAR2-R	TTCTCCCACAGTGATGCC	
VdANR-F	TGTTTGGATGAGTCCTGTTG	
VdANR-R	CACAAGCAAGGGTAACCACT	
VdUFGT-F	GATTGGAGTTTCAGGCATTCAAGGC	
VdUFGT-R	CATGCGTGAGAAGAGCGAGT	
α-Tubulin-F	GTTCTCGCGCATTGACCATA	
α-Tubulin-R	CAGCCAGATCTTCACGAGCTT	

Supplementary Sequence

Sequence S1:*VdLAR1*

>*VdLAR1* (GenBank: PQ185972.1) ORF 1-1041 bp

ATGACTGTTTCTCCGGTTCCTTCGCTCAAGGGTCGTGTCCTCATTGCCGGAGCAACCGGTTTCATTGGT
CAGTTCGTGGCCGCAGCAAGCCTTGATGCCCATCGACCCACCTACATTCTCGCACGTCCAGGCCCCAG
GAGTCCTTCTAAGGCCAAGATCATCAAGGCCCACGAGGACAAAGGCGCCATCATCGTATACGGGTTGA
TAAACGAGCAGGAGTCTATGGAGAAGATACTAAAAGAACATGAGATAGACATAGTAGTATCAACCGTG
GGCGGAGAGAGCATATTGGATCAAATCGCCCTAGTGAAAGCCATGAAGGCTGTTGGAACCATTAAGAG
ATTTTTGCCGTCTGAATTCGGGCACGATGTGAACAGAGCTGATCCAGTTGAGCCAGGGCTCAACATGT
ACAGAGAGAAGCGTAGGGTCCGACAATTTGTGGAGGAATCGGGCATACCCTTCACTTATATCTGCTGC
AACTCAATTGCTTCTTGGCCATACTACAATAACATTCACCCTTCTGAGGTTCTTCCTCCAACGGATTTCT
TCCAGATTTACGGTGATGGCAATGTCAAAGCTTACTTTGTTGCAGGCACAGACATCGGAAAATTCACG
ATGAAAACAGTGGACGATGTCCGAACACTGAACAAATCAGTGCATTTCCGGCCATCTTGCAATTGTCT
CAACATAAATGAACTCGCATCTGTGTGGGAAAAGAAGATTGGGAGGACACTTCCCAGAGTAACCGTC
ACTGAAGATGATCTACTAGCTGCAGCCGGAGAAAACATCATCCCACAGAGTGTGGTAGCGGCGTTCAC
GCACGACATTTTCATAAAGGGGTGTCAGGTGAATTTCTCTATTGATGGCCCGGAGGACGTGGAAGTGA
CCACCCTCTACCCTGAGGATTCTTTCAGGACGGTGGAGGAATGCTTCGGCGAATACATCGTGAAGATA
GAGGAAAAGCAGCCGACCGCCGATTCTGCTATTGCCAACACCGGTCCTGTGGTTGGGATGCGGCAAG

TCACTGCAACCTGCGCTTGA

Note: The start and stop codons are marked in red.
